# Factors Influencing Parents' Decision in Choosing a Pediatric Dentist

**DOI:** 10.7759/cureus.46812

**Published:** 2023-10-10

**Authors:** Shraddha P Kochar, Priyanka P Madhu, Amit R Reche, Bhairavi K Kale

**Affiliations:** 1 Public Health Dentistry, Datta Meghe Institute of Medical Sciences, Sharad Pawar Dental College, Wardha, IND; 2 Periodontics, Datta Meghe Institute of Medical Sciences, Sharad Pawar Dental College, Wardha, IND

**Keywords:** pediatric dentist, dental caries, parents knowledge, influencing factors, children

## Abstract

Introduction

Pediatric dentists support children's dental health while also providing parents with health education and information. Parents are guardians of their children's oral health, as their knowledge, beliefs, also attitude about oral health can influence early childhood oral health directly or indirectly. Recognizing parenting practices on early childhood oral health is essential for developing effective prevention and treatment strategies for the child, because parents' health attitudes, behavior, and their children's dental health are usually influenced directly or indirectly by their practice. Objectives of this study were to find out what factors parents prefer while choosing a pediatric dentist for their kids, to assess parent attitudes towards management of a symptomatic primary tooth, to analyze parental understanding of the importance of deciduous teeth, and to assess parent knowledge and perception regarding their child.

Methodology

A cross-sectional study conducted at Sharad Pawar Dental College and Hospital was done, where 20 questions were posed to the parents who came with their children for dental treatment. Questions were divided into two parts, knowledge-based assessment scale and perception-based assessment scale. The questions were formulated to assess the knowledge of parents regarding dental care, their perception, and their attitude toward the decision-making while selecting a pediatric dentist for their kids. The questions also included information about the child's habits and associated problems with it. Three hundred parents of children under 14 years of age were chosen randomly where all parents were adequately informed beforehand, and their written consent was taken before proceeding with the questions. All the questions were formulated in the native language that they could easily understand. A convenient method was applied as per the respondent from an offline questionnaire survey.

Results

By evaluating the parental knowledge as well as perception towards children's oral health and dental treatment we are able to determine which aspects parents prefer while selecting a pediatric dentist for their child. The study found that parents had limited knowledge about the causes, treatment, prevention, and consequences of oral health problems in children. Many parents neglect oral health problems and lack correct knowledge.

Conclusion

Hence this study assesses the parental knowledge as well as perception towards children's oral health. This study concludes that it is crucial to raise awareness about oral health among parents and inform them about available treatments, consequences of negligence and future problems to aid them in decision-making.

## Introduction

Oral health is now recognized as a critical component of overall health. As a result, the oral cavity is regarded as a "mirror" of general health. It is well known that a healthy mouth and body go hand in hand. Any deviation in oral health leads to poor oral health, which can lower an individual's quality of life and contribute to developing oral diseases. Even though oral problems are preventable, they remain a significant public health issue worldwide due to numerous barriers to oral care delivery and accessibility [1]. The prevalence of dental caries in primary teeth in children in the world with a sample size of 80,405 was 46.2%, and the prevalence of dental caries in permanent teeth in children in the world with a sample size of 1,454,871 was 53.8%. Therefore there is a need for awareness among parents and there is a need to educate them. Parents, not doctors, are the primary caretakers of their children’s health [2].

Pediatric dentistry is a young profession in India, and there is less awareness of it as a specialty than in Western nations, where it is well-established [3]. Indians have a lower level of oral health awareness and practice than Westerners. The belief that "Milk teeth aren't worth worrying about because they'll fall out anyhow" has all but disappeared in the Western world [4]. It has been said that Western children are more aware of the significance of regular dental visits due to their parents or dentists introducing them to the concept. In India, such effort on the part of the parents is almost non-existent [5]. The importance of dental checkups at a young age is neglected since most parents think their child's milk teeth will exfoliate, and it is not worth paying attention to [6]. Primary teeth play an essential role in the development of speaking, chewing, preserving space, and directing the emergence of permanent teeth during the first six to seven years of a child's life [7]. They also help in promoting self-confidence because kids who have decayed teeth usually do not smile or cover their mouth. A healthy smile gives children the self-confidence they need to have positive social experiences. Even if a parent is recommended to take their child to a pediatric dentist, many parents are ignorant of the importance of primary teeth. Parents' knowledge of dental treatment, and their attitudes, and behavior toward it substantially influence their children's development of a positive dental attitude [8]. There is mounting evidence that beginning oral health prevention early in life is beneficial. Preventing dental caries in very young children necessitates a significant investment by parents [9].

Primary dentition is possibly the most essential often underutilized of all the dentist's procedures [10]. Children's dentistry is referred to as pediatrics. Pediatric dentistry involves more than just dental expertise, since they are coping with individuals in their formative years [11]. Pedodontics is the precise term for this specialty service. The importance of pedodontic services cannot be overstated, because improper or insufficient dental care during childhood can permanently destroy the overall mastication apparatus (chewing structures), leaving the individual with a variety of dental disorders that haunt today's modern adult population [12]. It is widely acknowledged that parental behavior impacts their children's health. Parents' oral health knowledge, beliefs, and attitudes influence their children's health maintenance, dietary habits and health behavior [13].

## Materials and methods

After obtaining ethical approval from the Institutional Ethics Committee with approval number DMIMS(DU)/IEC/2022/972, Sharad Pawar Dental College and Hospital, Datta Meghe Institute of Medical Sciences (DMIMS), a descriptive, cross-sectional study was conducted among parents of 0-14 years of children who came with them in the hospital. The study was conducted for four months, from March 2022 to June 2022. Three hundred parents of children under 14 years of age were chosen randomly. This population was selected for study because it requires no extra time for parents to answer the questions as the study was conducted during waiting hours and parents' behavior plays a major role in child dentition. The sample size selected for this study is sufficient to make a conclusion. The data was collected from the parents who visited the pediatric dental clinic of Sharad Pawar Dental College and Hospital, Sawangi, Wardha for the dental treatment of their child and were requested to participate in the study. For analysis, the parents who voluntarily decided to take part in the study were included. Parents of kids with exceptional needs or accompanying systemic disorders were among those who were excluded and those parents who were not willing to participate. A questionnaire was designed for the study to be answered by parents. The questionnaire comprised 20 questions which were modified and finalized according to the responses from the parents. All the questions were written in the local language (Marathi), which was well understood by the parents, and, if and when needed, were translated for them. All respondents received guarantees of secrecy and anonymity. The parents were given an explanation of the study's goals and methodology in the language they were comfortable speaking. After receiving permission, the parents were taken to a waiting room, where they were asked to sign a consent form in their original mother tongue. After getting a signed agreement, the parents were questioned regarding their knowledge, opinions, and attitudes toward dental care and oral hygiene during the therapy period. The questionnaire was designed to help in the assessment of knowledge and perception of parents.

Statistical analysis

Statistical analysis was done by SPSS version 23 (IBM Inc., Armonk, NY, USA). Descriptive statistics and frequency distribution were done to determine the mean values and standard deviation. Chi-squared statistics was used to determine the association of demographic variables with the questionnaire. Statistical significance was kept at p<0.05.

## Results

Demographic details of the participants under study

The study had 300 parents who participated in the study, of which 293 were female and seven were male. The age group of the children ranged from two to 14 years, of which 100 children were two to four years old, 48 were five to seven years old, 71 were eight to 10 years old, 54 were 11 to 12 years old and 27 were 13 to 14 years old. One hundred forty-two children were male and 158 were female. One hundred twenty-seven parents had graduated, 31 were post-graduates and 142 non-graduates. Fifty-six parents had an income below 10000, 123 between 10000-100000, and 121 above 1 lakh (Table 1).

**Table 1:** Demographic variables of the participants under study (n=300)

Demographic variables		N	%	Mean	SD
Gender	Male	142	47	-	-
	Female	158	53	-	-
Age group	2-4 years	100	33	5.97	0.34
	5-7 years	48	16	5.21	0.30
	8-10 years	71	24	9.64	0.56
	11-12 years	54	18	10.52	0.61
	13-14 years	27	9	13.57	0.78
Parent along with child	Mother	293	97.6	-	-
	Father	7	2.3	-	-
Education Status of respondent	Graduated	127	42	-	-
	Post Graduated	31	10	-	-
	Non-Graduated	142	47	-	-
Income	Below 10000	56	18	-	-
	10000 - 100000	123	41	-	-
	Above 1 lakh	121	40	-	-

According to the study, only 24% (71) of parents were aware when the first tooth erupts, i.e. at six months. A statistically significant difference was observed in reference to age and gender. Twenty-nine percent (87) of parents were aware that the ideal kind of toothbrush for children is soft. Forty-two percent (125) of parents knew that tooth brushing is the method of teeth cleaning, 18% (55) said washing with water, 3% (10) said cleaning with powder and the remaining had no idea. Twenty-six percent (77) of parents were aware that teeth should be cleaned twice. A statistically significant difference was observed in reference to age and gender. Twenty-seven percent (82) of parents were aware that the correct method of tooth brushing for children is circular. Only 26% (79) of parents knew that toothbrushes should be changed every three months. A statistically significant difference was observed in reference to age and gender. Six percent (19) of parents had given their child sugary foods with meals, 44% (131) had given in between meals, 10% (30) had given before going to bed and 40% (120) were not particular. A statistically significant difference was observed in reference to age and gender. This study proves that parents commonly underestimate the amount of sugar in foods. The recommendation is to limit ‘added sugars’ to 6 teaspoons for children per day. Sugar tricks your body into thinking it needs more to eat. The largest risk of tooth decay is when sugar is consumed one hour before bed. Ten percent (31) of parents used dental floss as an additional cleaning aid, 0.3% (1) used toothpicks, 7% (21) used interdental brushes and 82% (218) had no idea regarding cleaning aids other than toothbrushes. A statistically significant difference was observed in reference to age and gender. Interdental cleaning aids are important for maintaining gingival health and avoiding oral disease. If teeth are tightly spaced, waxed floss or flossing tape glides between them more easily and helps in removing food particles trapped between teeth. Using a toothpick is avoided because it can increase risk of gum irritation and oral infection. Only 15% (46) of parents were aware that they should visit a dentist every six months. Only 11% (32) of parents were aware of the oral habits that can be harmful for the child (Table 2).

**Table 2:** Knowledge-based assessment scale

Questions		N (300)	%	P value (Gender, Age group)
1. Do you know when the first teeth erupt?	a. 1 month	7	2	0.001
	b. 6 month	71	24	
	c. 1 year	50	17	
	d. No idea	172	57	
2. Do you know the ideal kind of toothbrush for children?	a. Soft	87	29	0.210
	b. Medium	41	14	
	c. Hard	15	5	
	d. No idea	157	52	
3. What is the method for teeth cleaning you use at home?	a. Tooth brushing	125	42	0.070
	b. Washing with water	55	18	
	c. Cleaning with powder	10	3	
	d. No idea	110	37	
4. How many times per day should the teeth be cleaned?	a. Once	124	41	0.001
	b. Twice	77	26	
	c. After eating	10	3	
	d. No idea	89	30	
5. Which of the following is the correct method of teeth brushing?	a. Horizontal	65	22	0.055
	b. Circular	82	27	
	c. Horizontal with circular	12	4	
	d. No idea	141	47	
6. When should the toothbrush be changed?	a. After 1 month	28	9	0.001
	b. After 2 months	27	9	
	c. After 3 months	79	26	
	d. No idea	166	55	
7. When do you give your child sugary foods?	a. With Meals	19	6	0.001
	b. In between meals	131	44	
	c. Before going to bed	30	10	
	d. Not particular	120	40	
8. Other than toothbrushing, which cleaning aids do you use for your child's teeth cleaning?	a. Dental floss	31	10	0.001
	b. Toothpick	1	0.3	
	c. Interdental brush	21	7	
	d. No idea	248	82	
9. What is the suitable time to visit the dental clinic routinely?	a. Once every three months	15	5	0.041
	b. Once every 6 months	46	15	
	c. Once every year	21	7	
	d. No idea	218	73	
10. Are you aware of the oral habits that can be harmful for children?	a. Yes	32	11	0.210
	b. No	268	89	

According to the current study, only 8% (23) of parents visited the dentist with their child when their first tooth erupted. This means most of the parents were not aware of taking their child to the dentist as soon as their first tooth erupts. A statistically significant difference was observed in reference to age and gender. Fifty-six percent (169) of children had bad dental experiences, which means there is a need to provide better dental care to children and good communication skills. A statistically significant difference was observed in reference to age and gender. Sixty-two percent (186) parents were aware that there are dentists specially for children while 10% (29) had taken their children to a general dentist and 9% (27) had taken their children to the family doctor. The remaining parents had given home remedies to their children if they suffered from toothache. This means there is a need to make parents aware that there are dentists only for children.

According to the survey, 36% (108) of parents used a pacifier even after three to four years. Babies utilize pacifiers as a method of self-soothing. A misaligned bite is among the most well-known dangers associated with excessive pacifier use. A crossbite, an open bite, or other malocclusions may fall under this category. Particularly when older kids are still sucking on pacifiers, these oral problems are more frequently observed. Use of a pacifier for an extended period of time may cause a child's teeth to erupt and possibly alter the form of the roof of their mouth. A statistically significant difference was observed in reference to age and gender.

For 31% (94) of parents, dental clinic location was an important factor when choosing a pediatric dentist. These parents preferred a dental clinic that was close to their workplace or home and were not concerned about the experience or qualifications of the dentist. A statistically significant difference was observed in reference to age and gender. Eighty-three percent (248) of parents chose not to restore teeth if the child had caries. Such parents think that only when their child has pain they will take them to the dentist, otherwise there is no need to visit a dentist. Forty percent (119) of children were scared and reluctant when they visited a dentist for the first time. Nineteen percent (58) of children were slightly afraid and 18% (54) of children were very slightly afraid. The remaining children were never afraid and were very courageous and confident during the process of the dental treatment. This shows that dental procedures and instruments have a major impact on the behavior of the child in dental clinics. Eighty percent (240) of parents had taken their child to a dentist only when the child suffered from extreme pain. Only 13% (38) of parents had taken their child to a dentist once a year and 7% (21) of parents every six months. A statistically significant difference was observed in reference to age and gender. Eighty-seven percent (260) of parents thought that it is not important to take the child for regular dental check-ups.

Parents are the first sources of primary information that children receive about oral health. Thirty-six percent (109) of parents had poor dental health. Only 16% (48) of parents had good dental health and the remaining parents had fair dental health. Therefore it is important to educate parents about dental health and consequences of negligence. A statistically significant difference was observed in reference to age and gender (Table 3).

**Table 3:** Perception-based assessment scale

Questions		N (300)	%	P value
1. Did you visit the dentist when your child's first tooth erupted?	a. Yes	23	8	0.001
	b. No	277	92	
2. Have you or your children ever had a bad dental experience?	a. Yes	169	56	0.033
	b. No	131	44	
3. If your child suffers from toothache, to whom will you take them?	a. Family doctor	27	9	0.714
	b. General dentist	29	10	
	c. Paediatric dentist	186	62	
	d. Home remedy	58	19	
4. Does your child use a pacifier?	a. Yes	108	36	0.043
	b. No	192	64	
5. Is the location of a dental clinic a necessarily important variable when choosing a pedodontist?	a. Yes	94	31	0.001
	b. No	206	69	
6. If your child has caries, do you think it should be restored?	a. Yes	52	17	0.081
	b. No	248	83	
7. How was the behaviour of your child when you visited for the first time?	a. Scared and reluctant	119	40	0.781
	c. Very slightly afraid	58	19	
	c. Very slightly afraid	54	18	
	d. Never afraid	69	23	
8. How often do you take your child to a dentist?	a. Every 3 months	1	0.3	0.001
	b. Every 6 months	21	7	
	c. Once in year	38	13	
	d. Not particular	240	80	
9. Do you believe that it is important to take the child for regular dental visit?	a. Yes	40	13	0.540
	b. No	260	87	
10. How is your own dental health?	a. Good	48	16	0.001
	b. Fair	143	48	
	c. Poor	109	36	

## Discussion

Pediatric dentists make children's treatment less painful and treat them more effectively as compared to other dentists. They take extra measures to create a friendly, kind, and safe environment for their young patients. Aside from smaller dental equipment to treat children, they often have play areas in their waiting rooms or televisions with children's cartoons to make their patients feel at ease. Preventing primary tooth loss and minimizing the risk of subsequent decay in permanent teeth requires parents' oral health education and adequate oral hygiene practices. Jain and Gurunathan conducted a study on parents' views regarding various influencing factors on child behavior in dental clinics. This research demonstrates that a child's visit to the dentist is influenced by a variety of circumstances. Toothache was the most prevalent reason for a child's dental appointment, accounting for 54% of all visits. In this study, they conclude that most of the parents believe that the dental appointments, the attire of the dentist as well as the dental procedure have a major influence on the manner in which the child behaves in the dental clinic. The most significant component of a pediatric dentist's success is reliant not only on the technique used and the expertise possessed, but most crucially on the child and their parents. As a result, we would like to conclude that a child's behavior in the dental clinic is largely influenced by his or her parents' perceptions about dental treatment [14].

Mourad et al. conducted a study on factors influencing parents' decision for choosing a pediatric dentist at the University of Greifswald, Germany in 2015. A questionnaire with 30 elements that could potentially be important to decision-making was distributed to pediatric dentists across Germany to be delivered to new patients' parents (n=450). According to this study, previous dental visits by parents and children played a "significant" or "very important" impact in deciding on a pediatric dentist (78.8% and 62.2%, respectively). Friends and acquaintances were the most common source of referrals for a pediatric dentist (86.5%). Other dentists' recommendations were frequently included in decision-making (60.7%), whereas specialist journals or internet portals were less important to respondents (15% and 19%, respectively). The majority of the parents obtained information from internet search engines and the practice website [15].

Muzondo et al. conducted a similar investigation on determinants of parents’ choice of a pediatric dentist. The overall goal of this study was to uncover criteria that parents in Harare, Zimbabwe consider while selecting pedodontist services for their children. The ability to control uncooperative behavior, care and empathy, accessibility and convenience of the dental clinic, friendliness of supporting staff and general perspective of the dental environment, as well as the process by which dental therapy is conducted, all influence a pedodontist's choice. The study's main finding is that dentists should think about marketing as an important part of practice management [16].

The current study on factors influencing parents' decision in choosing a pediatric dentist concludes that previous dental experience, behavior of the child towards dental treatment and parent satisfaction are the important factors for parents for choosing a pediatric dentist for their children. According to this study, 24% of parents were aware when the first tooth erupts i.e. at six months, teeth should be cleaned twice, and toothbrushes should be changed after every three months. This study proves that parents commonly underestimate the amount of sugar in foods which is the main reason for caries. According to this study 92% of parents were not aware of taking their child to the dentist as soon as their first tooth erupts. Fifty-six percent of children had bad dental experience which means there is a need to provide better dental care to children and good communication skills. Thirty-one percent of parents preferred a dental clinic close to their workplace or home and were not concerned about the experience or qualifications of the dentist. Eighty-three percent of parents thought that only when the child has pain they will take them to the dentist otherwise there is no need to visit the dentist. Thirteen percent of parents had taken their child to the dentist once a year and 7% of parents every six months. In this study, we conclude that most of the parents are not aware of their children's oral health and their knowledge regarding dental health is not satisfactory.

This study had certain limitations. The study was done in a limited geographic area that did not relate to a wider population. This survey also lacks children's perspectives regarding dentists and dental treatment. It is important to understand children's perspective regarding dentists through their response to dental treatment and understand their fear towards dentistry and try to change their opinion for better treatment. But this study focuses only on parents' perceptions and it should be incorporated into future research.

## Conclusions

This research project provides insights into parental perspectives on the impact of children's oral health habits, as well as their views on what aid is required to promote children's dental health. According to this study most of the parents' knowledge regarding their children's dentition was not satisfactory. They were unaware of tooth cleaning methods and other aids, which has a negative impact on children's oral health. Previous dental experience, behavior of the child towards dental treatment, location of the clinic and parent satisfaction are the important factors for parents for choosing a pediatric dentist for their children. This study concludes that most parents are unaware that child dental specialists are there specially for children and neglect their oral condition as they are unaware about future complications. This study proves that parents commonly underestimate the amount of sugar in their children. According to the findings of this study, the majority of parents are unaware of their children's oral health, and their knowledge of dental health is inadequate. Therefore, there is a need for parental education and awareness among people about their child's dentition by conducting effective oral health programs and interventions. Children's dental checkup programs should be regularly conducted in schools and their opinions regarding dentistry should be changed through informative videos in a funny and interesting manner. The importance of healthy teeth should be included in their syllabus. Dental checkup camps should be conducted in rural areas and uneducated populations. Parents' views regarding children's dentition should be changed as it is equally important as permanent teeth.
